# The Peroxidatic Thiol of Peroxiredoxin 1 is Nitrosated by Nitrosoglutathione but Coordinates to the Dinitrosyl Iron Complex of Glutathione

**DOI:** 10.3390/antiox9040276

**Published:** 2020-03-25

**Authors:** Daniela R. Truzzi, Simone V. Alves, Luis E. S. Netto, Ohara Augusto

**Affiliations:** 1 Departamento de Bioquímica, Instituto de Química, Universidade de São Paulo, São Paulo 05508-000, Brazil; oaugusto@iq.usp.br; 2 Departamento de Genética e Biologia Evolutiva, Instituto de Biociências, Universidade de São Paulo, São Paulo 05508-090, Brazil; svidigal@ib.usp.br (S.V.A.); nettoles@ib.usp.br (L.E.S.N.)

**Keywords:** peroxiredoxin 1, nitrosoglutathione, dinitrosyl iron complex, kinetics, nitrosation, nitrosylation

## Abstract

Protein S-nitrosation is an important consequence of NO^●^·metabolism with implications in physiology and pathology. The mechanisms responsible for S-nitrosation in vivo remain debatable and kinetic data on protein S-nitrosation by different agents are limited. 2-Cys peroxiredoxins, in particular Prx1 and Prx2, were detected as being S-nitrosated in multiple mammalian cells under a variety of conditions. Here, we investigated the kinetics of Prx1 S-nitrosation by nitrosoglutathione (GSNO), a recognized biological nitrosating agent, and by the dinitrosyl-iron complex of glutathione (DNIC-GS; [Fe(NO)_2_(GS)_2_]^−^), a hypothetical nitrosating agent. Kinetics studies following the intrinsic fluorescence of Prx1 and its mutants (C83SC173S and C52S) were complemented by product analysis; all experiments were performed at pH 7.4 and 25 ℃. The results show GSNO-mediated nitrosation of Prx1 peroxidatic residue ($k_{+NO}^{Cys52}$ = 15.4 ± 0.4 M^−1^. s^−1^) and of Prx1 Cys^83^ residue ($k_{+NO}^{Cys83}$ = 1.7 ± 0.4 M^−1^. s^−1^). The reaction of nitrosated Prx1 with GSH was also monitored and provided a second-order rate constant for Prx1Cys^52^NO denitrosation of $k_{-NO}^{Cys52}$ = 14.4 ± 0.3 M^−1^. s^−1^. In contrast, the reaction of DNIC-GS with Prx1 did not nitrosate the enzyme but formed DNIC-Prx1 complexes. The peroxidatic Prx1 Cys was identified as the residue that more rapidly replaces the GS ligand from DNIC-GS ($k_{DNIC}^{Cys52}$ = 7.0 ± 0.4 M^−1^. s^−1^) to produce DNIC-Prx1 ([Fe(NO)_2_(GS)(Cys^52^-Prx1)]^−^). Altogether, the data showed that in addition to S-nitrosation, the Prx1 peroxidatic residue can replace the GS ligand from DNIC-GS, forming stable DNIC-Prx1, and both modifications disrupt important redox switches.

## 1. Introduction

Protein S-nitrosation (P-CysNO), extensively named S-nitrosylation, is a post-translational modification involving the covalent addition of a nitrosonium (NO^+^) to the sulfur atom of a deprotonated protein cysteine residue (P-CysS^−^). Numerous studies have shown that protein S-nitrosation is a ubiquitous and reversible post-translational modification to which cytotoxic and signaling properties have been attributed [1,2,3], although the latter have been recently challenged [4]. Protein S-nitrosation results from NO^●^ metabolism by routes that remain unclear and different possibilities have been proposed. The most popular are the transnitrosation reactions, in which the NO^+^ of a nitrosothiol is transferred to a protein thiolate (P-CysS^−^). Also, radical mechanisms involving either the oxidation of NO^●^ to NO_2_^●^ followed by their recombination to produce the nitrosating agent N_2_O_3_ or recombination of NO^●^ with low-molecular-weight thiyl radicals or protein-cysteinyl radicals (P-CysS^●^) (reviewed in [5,6]). However, radical reactions are usually rapid but have low specificity, whereas transnitrosation reactions are commonly slow and require a nitrosothiol precursor. Therefore, alternative pathways involving nitrosyl heme [7,8] or dinitrosyl-iron complexes (DNICs) [9,10] were also proposed. The latter possibility is particularly interesting because DNICs are consistently detected in cells overproducing NO^●^ and are considered the major cellular NO^●^-metabolite [11]. All of these pathways are under active investigation, particularly in cell culture experiments. Nevertheless, kinetic data on protein S-nitrosation by different agents are limited and the capability of DNICs in promoting protein S-nitrosation has yet to be investigated.

Peroxiredoxins (Prxs) are a family of abundant Cys-dependent peroxidases that react rapidly with peroxides, constituting an important antioxidant defense and acting as sensors and transmitters of H_2_O_2_ signals in cells [12,13,14]. There are six human Prxs (Prx1 to Prx6) that differ in their intracellular location and catalytic mechanisms [15]. Prx1 to Prx4 are typical 2-Cys Prxs that, in the reduced state, assemble into a decameric/duodecameric toroid, which is the most active form of the enzyme [16,17]. The minimal functional unit for 2-Cys Prxs is a homodimer and its peroxidase activity depends on a fully conserved Cys residue that has a low pK_a_ and is known as the peroxidatic cysteine (C_P_SH). This residue rapidly reacts with peroxides, reducing them while being oxidized to the sulfenic acid derivative (C_P_SOH), which can condense with the resolving Cys (C_R_SH) residue of the adjacent monomer, forming a head-to-tail disulfide (C_P_S-SC_R_). This Prx intermolecular disulfide is reduced by the thioredoxin system, turning over the peroxidase cycle of these enzymes. However, the condensation reaction is relatively slow, especially for eukariotic 2-Cys Prxs, and excess oxidant can further oxidize C_P_SOH to the sulfinic (C_P_SO_2_^−^) and sulfonic (C_P_SO_3_^−^) acid-derivatives. This process is called hyperoxidation and leads to the inactivation of the Prx peroxidase activity since C_P_SO_2_^−^ and C_P_SO_3_^−^ cannot be reduced by classical biological reductants, such as thioredoxin [12,13,18]. Upon the hyperoxidation of 2-Cys-Prxs, the antioxidant and redox relay functions of the enzymes decline but other actions may rise, such as the chaperone-like activity, potentiation of redox signaling pathways mediated by Cys-based proteins that are poorly reactive towards H_2_O_2_ and maintenance of Trx-dependent activities (reviewed in [19]).

The high cellular abundance of Prxs and the low pK_a_ of their peroxidatic Cys residue make these enzymes potential targets of S-nitrosation. Indeed, several members of the Prxs family, especially Prx1 and Prx2, were detected as being S-nitrosated in multiple mammalian cell types under a variety of treatments [20,21,22,23,24]. Depending on cell type and experimental settings, Prx1 and/or Prx2 nitrosation was reported to decrease the peroxidase activity of the enzymes [23,25], protect them from hyperoxidation [22] and stimulate their chaperone-like activity [24]. These cellular studies point to a relevant influence of Prx1 and Prx2 S-nitrosation on the antioxidant and signaling functions of these enzymes.

As is the case of other proteins, there is a lack of kinetic studies on Prxs S-nitrosation by different agents. A previous investigation with recombinant human Prx1 reported that the enzyme is readily transnitrosated by nitrosocysteine (CysNO) and by nitrosogluthatione (GSNO) in vitro and in cells with consequent structural and functional alterations [23]. These authors focused mainly on CysNO as the S-nitrosating agent and presented quite limited kinetic data. Here, we investigated the kinetics of Prx1 S-nitrosation by GSNO, a recognized biological nitrosating agent [26], and by the DNIC complex of glutathione (DNIC-GS), a hypothetical nitrosating agent [9,10]. Our results show that GSNO-mediated nitrosation of Prx1 peroxidatic Cys is a quite slow process ($k_{+NO}^{Cys52}$ = 15.4 ± 0.4 M^−1^. s^−1^) and is efficiently reversed by glutathione (GSH) ($k_{-NO}^{Cys52}$ = 14.4 ± 0.3 M^−1^. s^−1^). In contrast, the Prx1 peroxidatic Cys is not nitrosated by DNIC-GS but displaces the thiol ligand, forming a DNIC-Prx1 complex.

## 2. Materials and Methods

All chemicals were purchased from Sigma-Aldrich, Merck or Fisher and were analytical grade or better. The H_2_O_2_ solutions were prepared from stock immediately before use and their concentrations were determined spectrophotometrically at 403 nm (ε = 54,000 M^−1^. cm^−1^) by reaction with horseradish peroxidase to produce compound I [27]. Solutions of GSNO were freshly prepared, kept on ice and protected from light [5]. The GSNO concentration was determined at 355 nm (ε = 922 M^−1^. cm^−1^) [5]. All solutions and buffers were prepared with Milli-Q water (Millipore).

### 2.1. Expression and Purification of Recombinant Proteins

Human Prx1 (wild-type, C52S, and C83SC173S mutants) was cloned into pET-17b [23,28] while human Prx2 was cloned into pET28a [29], both were expressed in *E. coli* BL21 cells and purified as previously described. Prx1 wild-type and C52S and C83SC173S mutants were purified using an anionic Exchange MonoQ column (GE Healthcare) coupled to the FPLC (AKTA, GE Healthcare, General Electric Co., Chalfont St. Giles, United Kingdom) with the automatic collector Frac-900. The buffer used was 25 mM Tris, 1 mM EDTA, 2 mM DTT, pH 8.8, glycerol 10% and the mobile phase gradient ranged from 0 to 1 M NaCl. Prx1 eluted in the unbound material. The purification of His-tagged Prx2 was performed with a HisTrap HP cobalt column (GE Healthcare). The coupling buffer used was 20 mM Tris-HCl pH 7.4, 250 mM NaCl and 10 mM imidazole and the elution buffer 20 mM Tris-HCl pH 7.4, 250 mM NaCl and the imidazole gradient ranged from 10 to 250 mM. The protein purity was evaluated by SDS-PAGE. The fractions with a higher degree of purity were combined and concentrated by ultrafiltration (cut-off filter 10-kDa, Amicon Ultra, Millipore). *S. cerevisiae* thioredoxin 1 (Trx1) was cloned into pET-17b, expressed in *E. coli* strain BL21 and purified as previously described [23,30]. The Trx1 was purified in a Mono Q column (GE Healthcare; Chicago, IL, USA.) equilibrated with Tris-HCl (20 mM), pH 7.4. The proteins were eluted with a rising gradient of 0 to 1M NaCl. Trx1 eluted at approximately 20% NaCl. The collected fractions were analyzed by SDS-PAGE and those with high Trx1 purity were pooled and concentrated on an Amicon filter (3 kDa) with buffer exchange to phosphate buffer (20 mM), pH 7.4 and maintained in the refrigerator. His-tagged *S. cerevisiae* thioredoxin reductase 1 (TrxR1) was cloned into pPROEX-1, expressed in *E. coli* DH5 strain and purified as previously described [31]. After chromatography, the fractions containing high purity Trx1 were selected by SDS-PAGE, pooled together and dialyzed against phosphate buffer (20 mM), pH 7.4. The dialyzed sample was concentrated on an Amicon filter (3 kDa) and maintained in the refrigerator. The concentration of the proteins was determined at 280 nm using the ε values provided by the ProtParam tool [32].

### 2.2. Prx1 and Prx2 Thiol Reduction

Prx1 and Prx2 were reduced prior to use with a 20-fold molar excess of DTT under argon atmosphere during 2 h at 37 °C. The excess of DTT was removed by ultracentrifugation using cut-off filter 10 kDa (Amicon Ultra, Millipore) at 5 °C. In general, the concentration of the proteins was determined at 280 nm using the absorptive coefficient provided by ProtParam tool [32]. An exception was the protein pre-treated with DNIC, in which case the protein concentration was determined using the Bradford method because DNIC presents an absorption band at 280 nm.

### 2.3. Quantification of Prx1 Thiol Groups

Reduced wild-type, mutants or modified Prx1 were treated with excess of DTDP (4,4′−dithiodipyridine) (0.5 mM) for 15 min in 50 mM sodium phosphate pH 7.4, 150 mM NaCl, 0.1 mM DTPA (diethylenetriaminepentaacetic acid), and 0.1% SDS. Then, thiol quantification was performed spectrophotometrically at 324 nm (ε = 19,800 M^−1^. cm^−1^) [33]. The thiol contents from reduced wild-type Prx1, Prx1C52S and Prx1C83SC173S were typically 3.7 ± 0.3, 2.5 ± 0.2 and 1.8 ± 0.3 thiols/protein, respectively. Freshly reduced Prx1 was used in all experiments.

### 2.4. Quantification of NO^●^ and NO^●^-Derived Products

The quantification of NO^●^-derived products in Prx1 samples was performed by gas phase chemiluminescence using a Nitric Oxide Analyzer or the Saville–Griess assay. For both methods, before analysis, the Prx1 pre-treated with GSNO or DNIC-GS was washed exhaustively through five consecutive centrifugations (~30 min at 5 °C) using cut-off filters 10 kDa (Amicon Ultra, Millipore, Burlington, USA) and a washing buffer volume twenty times higher than the initial sample volume. The gas-phase chemiluminescence analysis was performed as optimized by Feelisch and collaborators [34]. A vessel containing a solution of 45 mM KI and 10 mM I_2_ in glacial acetic acid was maintained at 60 °C under a continuous nitrogen purge. Then protein samples were injected into the vessel directly or after incubation with 1% sulfanilamide in 5% HCl to trap NO_2_^−^ or with 1% sulfanilamide in 5% HCl plus 0.2% HgCl_2_ to trap NO_2_^−^ and decompose nitrosothiols [34]. For Prx1 pre-treated with DNIC, the treatment with HgCl_2_ was not performed because it accelerates DNIC degradation resulting in an overestimation of nitrosothiol quantification [35]. Calibration curves were constructed using standard NO_2_^−^ solutions under the same experimental conditions. In the Saville–Griess assay, Prx1 pre-treated with GSNO was incubated to a final volume of 300 μL in the assay buffer (1% sulfanilamide, 0.1% *N*-(1-naphthyl)ethylenediamine dihydrochloride, 5% HCl) in the absence or presence of 0.2% HgCl_2_ for 30 min and the absorbance monitored at 540 nm [36]. Calibration curves were constructed using standard NO_2_^−^ solutions.

### 2.5. Kinetic Studies of Prx1 S-Nitrosation by GSNO

The changes in the intrinsic fluorescence of freshly reduced wild-type Prx1 or Prx1C83SC173S upon GSNO addition were monitored in a Hitachi F-2500 fluorimeter (Hitachi High Technology America, Inc.) using an excitation wavelength of 280 nm and an emission wavelength of 330 nm. All experiments were performed in phosphate buffer (50 mM) containing DTPA (0.1 mM), pH 7.4, at 25 °C. OriginPro 8.0 software was used for kinetic data analysis. Data from Prx1C83SC173S were fitted to a single-exponential function to obtain the pseudo-first-order rate constant (*k*_obs_) values. Data from wild-type Prx1 were fitted to a double-exponential function in which the second exponential was fixed to the *k*_obs_ value of the single-exponential fit of the reaction between Prx1C83SC173S and GSNO. The apparent second-order rate constants were determined from the slope of *k*_obs_ values plotted against GSNO concentrations using linear least-square regression analysis.

### 2.6. Kinetic Studies of Prx1 S-Denitrosation by GSH

Reduced wild-type Prx1 and Prx1C83SC173S were treated with 80-fold excess of GSNO over protein concentration for 1 h and 30 min, respectively, at 25 °C. Then, the treated proteins were transferred to ice and washed exhaustively (~30 min) using cut-off filters 10 kDa (Amicon Ultra, Millipore) at 5 °C to remove the low molecular weight compounds. The final S-nitrosated proteins were quantified and the number of modified thiols determined by DTDP as described above. The treated proteins were used only if thiol contents were around 1 thiol/protein in Prx1C83SC173S and 1–1.5 thiols/protein in wild-type Prx1. The changes in the intrinsic fluorescence of these S-nitrosated proteins upon GSH addition were monitored in a Hitachi F-2500 fluorimeter (λ_exc_ = 280 nm; λ_em_ = 330 nm) or in a Applied Photophysics SX-18MV stopped-flow spectrometer (λ_exc_ = 280 nm; λ_em_ > 320 nm). All experiments were performed in phosphate buffer (50 mM) containing DTPA (0.1 mM), pH 7.4, at 25 °C. The *k*_obs_ values were determined by fitting the traces to a single-exponential function. The apparent second-order rate constants were determined from the slope of *k*_obs_ values plotted against GSH concentrations, using linear least-square regression analysis.

### 2.7. Prx1 Thiol Alkylation and Prx1 His Ethoxyformylation

Alkylation of wild-type Prx1 thiols was obtained by incubating reduced Prx1 with a 100-fold excess of NEM (N-ethylmaleimide) [37] or 40-fold excess of IAM (iodoacetamide) [38] in the dark for 3 and 1 h, respectively. The excess of NEM or IAM was removed by ultrafiltration cut-off filter 10 kDa (Amicon Ultra, Millipore) at 5 °C. To modify Prx1 His residues, the enzyme Prx1 was treated with a 40-fold excess of DEP (diethylpyrocarbonate) at pH 5.0 (acetate buffer) for 1h30min (at pH lower than 6.0 the reaction is quite selective for His) [39,40]. The excess of DEP was washed away by ultrafiltration (cut-off filter 10 kDa). The ethoxyformylation of His residues was monitored by differential UV spectra at 242 nm (ε = 3200 M^−1^. cm^−1^) [39,40]. After His modification, the modified Prx1 was reduced (20-fold molar excess of DTT) before use.

### 2.8. Prx1 Peroxidase Activity Assay

Samples of reduced wild-type Prx1 (60 μM) were incubated with 60 or 120 μM of DNIC-GS for 30 min at 25 °C. Then, the low molecular weight molecules were washed away using a cut-off filter 10 kDa (Amicon Ultra, Millipore) at 5 °C. As DNICs exhibit absorption at 280 nm, untreated Prx1 or that treated with DNIC-GS were quantified by the Bradford method. The peroxidase activity was monitored by NADPH (100 μM) oxidation coupled to H_2_O_2_ (200 μM) reduction sustained by Prx1 (0.5 μM)/Trx1 (3 μM)/TrxR1 (1.5 μM) followed spectrophotometrically at 30 °C (NADPH; ε_340nm_ = 6220 M^−1^. cm^−1^) [41]. This assay was performed in 50 mM Hepes-NaOH at pH 7.0, and Trx1 and TrxR1 reductase were from yeast.

### 2.9. Dinitrosyl Iron Complex of GSH (DNIC-GS) Synthesis

The DNIC-GS was synthesized according to the literature [42]. Briefly, 28 mg of FeSO_4_ × 7H_2_O (10 mM), 68 mg of GSH (10 mM) and 6.9 mg of NaNO_2_ (10 mM) were added to 10 mL of degassed water. After 1 h, the solution pH was increased to 7.2 by NaOH addition. The reaction proceeded under an argon atmosphere and room temperature for 5 h. Then, the solution was filtrated for removal of iron hydroxide complexes and the DNIC was quantified by absorption bands at 310 and 360 nm (ε = 9200 and 7400 M^−1^. cm^−1^, respectively). Under these conditions, about 95–93% of the iron is present in the binuclear form (B-DNIC-GS) and 5–7% is present in the mononuclear form (M-DNIC-GS) [42].

### 2.10. Kinetics of Conversion of B-DNIC-GS into M-DNIC-GS

The conversion of B-DNIC-GS into M-DNIC was followed by absorption at 400 nm [42,43] in an Applied Photophysics SX-18MV stopped-flow spectrometer. Solutions of pre-synthesized DNIC-GS containing mostly B-DNIC-GS (95–93%) were mixed with an excess of GSH (final concentrations of 0.3 mM DNIC-GS and 4–26 mM GSH) in a phosphate buffer at pH 7.4 (200 mM) at 25 °C. The increase in absorbance was fitted to a single-exponential function. Then, the slope of the plot of *k*_obs_ values against GSH concentration provided the second-order rate constant.

### 2.11. Electon Paramagnetic Ressonance (EPR)

EPR spectra were recorded using a Bruker EMX spectrometer equipped with a high sensitivity cavity. The scanning at room temperature was performed with a flat cell and the EPR instrument operated at 9.85 GHz microwave frequency, 150 G range, 2 G amplitude modulation, 163.84 ms time constant and microwave power 20 mW. The spectra shown are the average of 8 scans. For the experiments at 77 K, the samples in 20% glycerol were frozen in a quartz tube (5mm inner diameter) and introduced into a quartz Dewar containing liquid nitrogen. The EPR instrument operated at 9.49 GHz microwave frequency, 300 G range, 4 G amplitude modulation, 81.92 ms time constant and 10 mW microwave power. The spectra shown are the average of 4 scans. The concentration of the paramagnetic species was calculated by double integration of the EPR signal compared to standard curves of known concentrations of 4-hydroxy-2,2,6,6-tetramethyl-piperidine-1-oxyl (tempol) or M-DNIC. For the kinetics of the reaction between wild-type Prx1 and DNIC-GS, a fixed concentration of DNIC-GS (60 μM) was mixed with Prx1 (60–180 μM) promptly transferred to a flat cell and the acquisition started. After reagent mixture (*t* = 0), all the procedure (transfer to the cell, to the instrument and spectrum scanning) time was considered in data processing. The plot of the EPR signal area against time was fitted to a single-exponential function. The apparent second-order rate constant was determined from the slope of the *k*_obs_ values plotted against the Prx1 concentrations, using linear least-square regression analysis. All experiments were performed in phosphate buffer (50 mM) containing DTPA (0.1 mM), pH 7.4, at 25 °C.

## 3. Results

### 3.1. Kinetics and Products of the Reaction of Prx1 with GSNO

**Figure 1:**
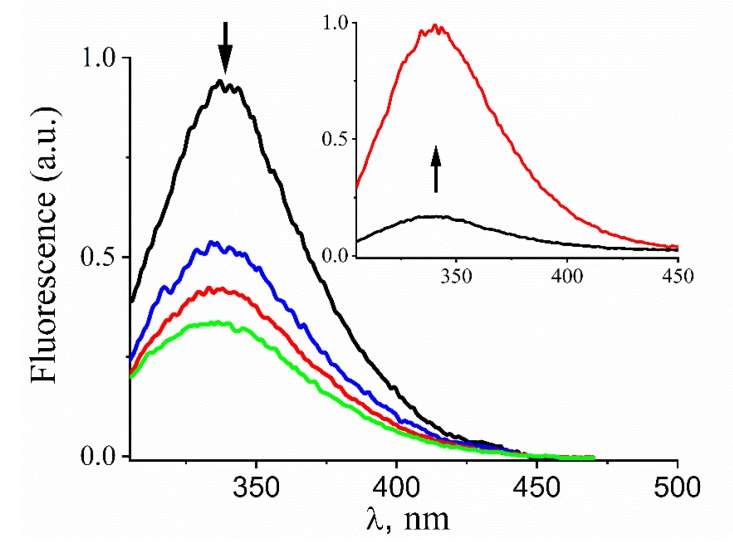
Changes in the intrinsic fluorescence of Prx1C83SC173S upon S-nitrosation and denitrosation. Representative emission spectra (λ_exc._ = 280 nm) of reduced Prx1C83SC173S (5 µM) before (black trace) and 2 (blue trace), 5 (red trace), and 10 min (green trace) after addition of GSNO (nitrosoglutathione) (400 µM). Inset: representative emission spectra (λ_exc._ = 280 nm) of S-nitrosated Prx1C83SC173S (5 µM) before (black trace) and 10 min (red trace) after addition of glutathione (400 µM). The rows indicate the temporal spectral changes. The incubations were performed in phosphate buffer (50 mM) containing DTPA (diethylenetriaminepentaacetic acid) (0.1 mM), pH 7.4 and 25 °C.

Changes in the intrinsic fluorescence of proteins upon their modification by oxidants and nitrosating agents have been extensively used to perform kinetic investigations, such as the kinetics of Prx1 oxidation by peroxides [44,45,46] and of glutathione S-transferase S-nitrosation by GSNO [47]. Therefore, we started by treating the mutant Prx1C83SC173S (5 μM) with GSNO (400 μM) and examined the changes in its intrinsic fluorescence. This mutant was selected because it contains the peroxidatic (Cys^52^) but not the resolving (Cys^173^) or the Cys^83^ residue, which were shown to be Prx1 targets for nitrosation by mass spectrometric experiments with cells and with Prx 1 treated with CysNO [22,23]. As shown in Figure 1, the intensity of reduced Prx1C83SC173S fluorescence decreased upon the addition of GSNO in a time-dependent manner. The process was reversible because the enzyme treated with GSNO for 30 min, purified from excess GSNO/GSH excess (cut-off filters) and quantified, recovered the initial fluorescence upon GSH 400 μM addition (Figure 1, Inset). These results confirm that the intrinsic Prx1 fluorescence can be used to follow S-nitrosation of Prx1 and suggest that treatment of Prx1C83SC173S with GSNO leads to Prx1C83SC173S nitrosation, which is reversed (denitrosated) by GSH addition.

To substantiate the above suggestion, we determined the thiol contents of Prx1C83SC173S before and after reaction with an excess of GSNO (80-fold) for 30 min at pH 7.4, 25 °C. Reduced Prx1C83SC173S usually contained 1.8 ± 0.2 thiol/protein while the GSNO-treated enzyme presented 1.0 ± 0.2 thiol/protein (Table 1). Therefore, approximately 1.0 thiol/protein disappeared after GSNO treatment. To test if this disappearance corresponded to S-nitrosation, treated Prx1C83SC173S was thoroughly washed in cut-off filters (10 kDa) and analyzed for NO^●^−derived products with a chemiluminescent analyzer, as previously optimized [34]. The sample was divided into two aliquots and, before analysis, one aliquot was treated with sulfanilamide/H^+^ (to trap NO_2_^−^) and the other aliquot with sulfanilamide/H^+^ plus HgCl_2_ (to decompose nitrosothiols). The aliquot pretreated with sulfanilamide/H^+^ showed a peak, the area of which corresponded to 0.73 ± 0.02 NO^●^/protein whereas the other aliquot did not show a peak. These experiments indicate that all NO^●^ detected in the GSNO-treated sample corresponded to nitrosated Prx1C83SC173S. These results were qualitatively and quantitatively confirmed by parallel experiments using the method of Saville [48] (data not shown). Taken together, these data indicate that the peroxidatic residue (Cys^52^) of Prx1C83SC173S Cys residue is nitrosated by GSNO.

**Figure 2:**
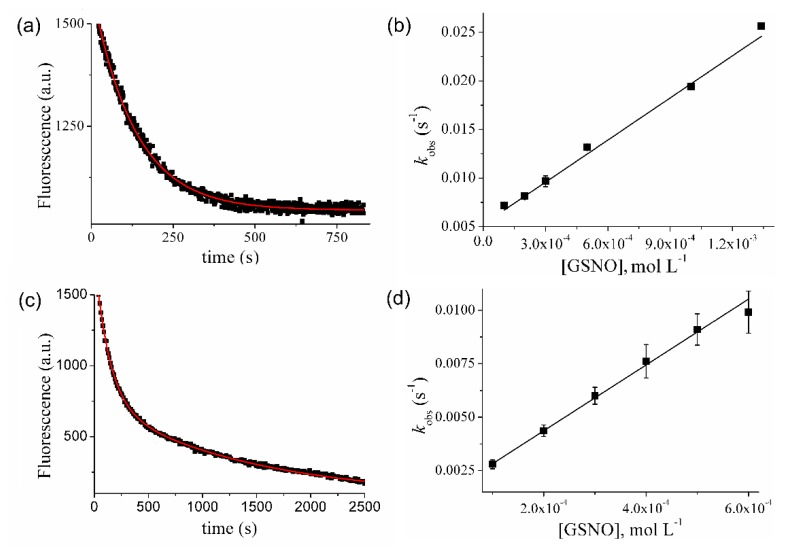
Kinetics of GSNO-mediated nitrosation of Prx1C83SC173S and of Prx1. (**a**) Representative kinetics of reduced Prx1C83SC173S (5 µM) S-nitrosation by GSNO (400 μM) (λ_exc._ = 280 nm; λ_em._ = 330 nm). The red trace corresponds to the fitting of the data to a single-exponential function. (**b**) Determination of the second-order rate constant of the reaction of Prx1 catalytic cysteine (Cys^52^) with GSNO. Pseudo-first-order rate constants (*k*_obs_) were plotted against GSNO concentration and the second-order rate constant obtained from the slope. (**c**) Representative kinetics of reduced Prx1 (5 µM) S-nitrosation by GSNO (400 μM). The red trace corresponds to the fitting of the data to a double-exponential function. (**d**) Determination of the second-order rate constant of the reaction of wild type Prx1 Cys^83^ with GSNO. Pseudo-first-order rate constants (*k*_obs_) for the second-exponential fluorescence decay were obtained by fixing the first-exponential fluorescence decay to the *k*_obs_ value obtained from the Prx1C83SC173S and GSNO reaction. Then, the *k*_obs_ values were plotted against GSNO concentration and the second-order rate constant obtained from the slope. All the experiments were performed using a Hitachi F-2500 fluorimeter in phosphate buffer (50 mM) containing DTPA (0.1 mM), pH 7.4, at 25 °C.

Next, we performed kinetic experiments following the decay of the maximum Prx1C83SC173S (5 μM) fluorescence (330 nm) with time under pseudo-first-order conditions. As anticipated, the rate of fluorescence decay with time increased with GSNO concentration (0.1 to 0.6 mM) and fitted to a single-exponential function (Figure 2a) from which the *k*_obs_ values were calculated. The plot of these *k*_obs_ values vs. GSNO concentration showed a linear dependency (Figure 2b) and the slope provided the second-order rate constant value of the reaction between GSNO and the peroxidatic residue (Cys^52^) of Prx1C83SC173S as k = (15.4 ± 0.4) M^−1^. s^−1^ at pH 7.4 and 25 °C.

Next, we investigated the reaction between reduced wild type Prx1 (referred to as Prx1) (5 μM) and GSNO (0.4–2.0 mM). In this case, the intensity of the intrinsic enzyme fluorescence also decreased with time after mixing with GSNO, but the decay followed a double-exponential function (Figure 2c). The rate of both decays was dependent on GSNO concentration, suggesting S-nitrosation of two Cys residues. The decays were fitted to a double-exponential function in which both exponents were allowed to float. The *k*_obs_ obtained for the first-exponential decay was quite similar to the one obtained for Prx1C83SC173S, indicating the same process. To obtain a more accurate value for the second-exponential decay, the first exponent was fixed to the experimental *k*_obs_ values determined for the reaction between Prx1C83SC173S and GSNO. Using this approach, we obtained excellent fits for the experimental data (as exemplified in Figure 2c). The obtained *k*_obs_ values for the slower decay were plotted against GSNO concentration (Figure 2d) and the slope provided the second-order rate constant value of k = (1.7 ± 0.4) M^−1^. s^−1^ at pH 7.4 and 25 °C for S-nitrosation of a second Cys residue.

**Figure 3:**
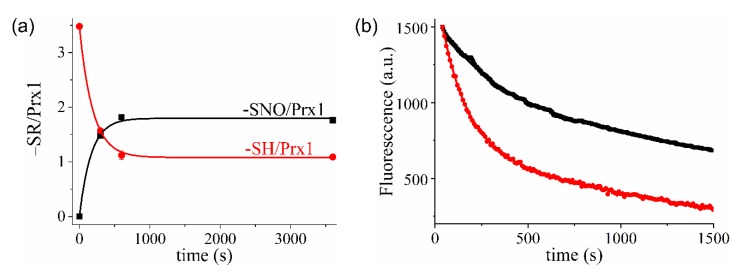
GSNO promotes S-nitrosation of two cysteine residues in wild-type Prx1. (**a**) Time-dependent content of thiol/protein (red trace) and nitrosothiol/protein (black trace) in Prx1 during its reaction with a 80-fold molar excess of GSNO (400 μM) (**b**) Representative kinetics of the reaction between reduced (red curve) or oxidized (black curve) Prx1 (5 μM) with GSNO (400 μM) at pH 7.4 and 25 °C (λ_exc._ = 280 nm; λ_em._ = 330 nm, Hitachi F-2500 fluorimeter).

Reduced Prx1 usually contained 3.5 ± 0.3 thiol/protein (Table 1). Addition of GSNO (80-fold the protein concentration) led to a time-dependent decrease in thiol/protein reaching a plateau of 1.1 ± 0.2 thiols/protein (Figure 3a). Simultaneously, an increase in the nitrosothiol content of up to 1.8 ± 0.3 nitrosothiol/protein was observed (Table 1). Fitting Prx1 nitrosothiol formation as a function of time to a single-exponential function provided a *k*_obs_ value of 6 × 10^−3^ s^−1^. This value is in good agreement with the *k*_obs_ calculated from fluorescence decay for the more rapid reaction under the same conditions (*k*_obs_ = 7.6 × 10^−3^ s^−1^) (Figure 3b, inset). Nitrosation of the second residue was not evidenced by this method, probably due to its low sensitivity as compared to fluorescence. At the end of the reaction, Prx1 exhibited 2.4 ± 0.3 modified thiol/protein, from which 1.8 ± 0.3 were identified as S-nitrosothiol (Table 1). Even though this difference is apparently within the experimental error, it likely indicates the formation of Prx1 disulfide bonds. An increase in dimer formation during the reaction between Prx1 and CysNO in a time-dependent manner has been reported [23]. We repeated these experiments, replacing CysNO with GSNO, and observed an increase in Prx1 dimers compared to control (Figure S1). However, no time dependence on dimer formation was observed, although oligomer formation showed a marginal time-dependent increase. Prx1 oxidation is extremely rapid and occurs even with contaminant H_2_O_2_ from buffers [49] while the reaction of Prx1 with GSNO is quite slow (Figure 2). Therefore, most of the Prx1 oxidation in the presence of GSNO occurred before S-nitrosation (Figure S1).

The data presented up to this point supported S-nitrosation of two Cys residues in Prx1 by GSNO and allowed assignment of the faster reaction to Prx1-Cys^52^NO formation. The identity of the second residue undergoing S-nitrosation remained unaddressed here although a previous mass spectrometric study of CysNO-treated Prx1 indicated Cys^83^ as the secondary nitrosation target [23]. To confirm that the same was true with GSNO, reduced Prx1 was oxidized with an equimolar concentration of H_2_O_2_ for 10 min to form the specific disulfide bond formation between catalytic and resolving cysteine (Prx1-Cys^52^-Cys^173^-Prx1). Then, the reaction between oxidized Prx1 (5 μM) and GSNO (400uM) was followed by fluorescence. As shown in Figure 3b, the first-exponential decay was considerably decreased by Prx1 oxidation while the second-exponential decay was unaffected. These experiments indicated that the resolving cysteine residue (Cys^173^) does not participate in the fluorescence changes observed during the reaction of Prx1 with GSNO and that the second-exponential decay corresponds to Cys^83^NO formation ($k_{+NO}^{Cys83}$ = (1.7 ± 0.4) M^−1^. s^−1^ at pH 7.4 and 25 °C).

### 3.2. Kinetics of Prx1 S-Denitrosation

**Figure 4:**
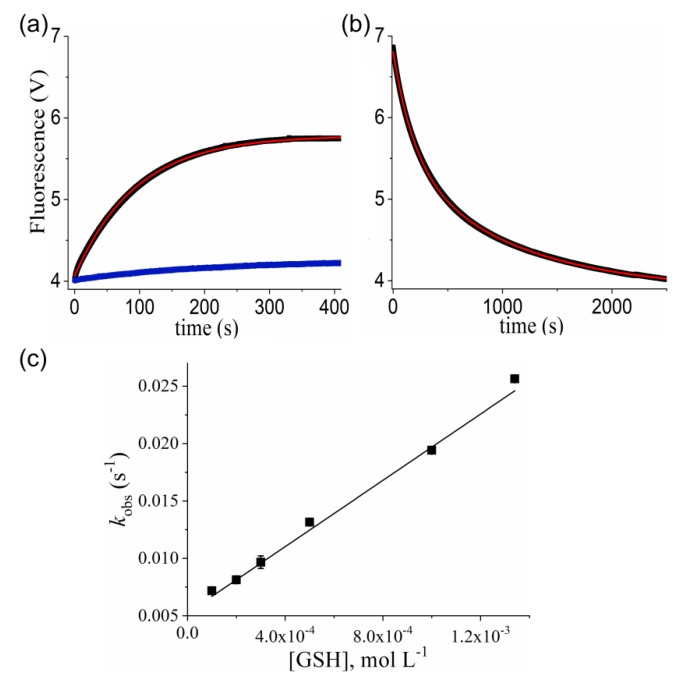
Kinetics of S-denitrosation of Prx1 by GSH. (**a**) Representative kinetics of spontaneous denitrosation (blue trace) and denitrosation mediated by GSH (400 μM) (black trace) for nitrosated Prx1 (5 µM); (**b**) representative kinetics of reduced wild-type Prx1 (5 µM) nitrosation by GSNO (400 μM) (λ_exc_ = 280 nm; λ_em_ > 320 nm) (Photophysics SX-18MV stopped-flow spectrometer). The red trace corresponds to the fitting of the data to a double-exponential function (right panel) and to a single-exponential function (left panel). (**c**) Determination of the second-order rate constant of the reaction of Prx1-Cys^52^NO with GSH. The determined pseudo-first-order rate constants (*k*_obs_) were plotted against GSH concentration and the second-order rate constant obtained from the slope. All the experiments were performed phosphate buffer (50 mM) containing DTPA (0.1 mM), pH 7.4, at 25 °C.

Prx1 treated with GSNO was immediately washed with cut-off filters, quantified and used to follow enzyme S-denitrosation by changes in the intrinsic fluorescence of the enzyme at pH 7.4 and 25 °C. The spontaneous (Figure 4a, blue trace) and GSH-mediated S-denitrosation followed (Figure 4a, black trace). The fitting of the spontaneous increasing of the intrinsic of fluorescence of S-nitrosated Prx1 (5 μM) to a linear function gave a $k_{-NO}^{}$ = (7.5 ± 0.5) × 10^−4^ s^−1^ (t_1/2_ = 15.4 min). Addition of GSH (0.1–1.34 mM) to S-nitrosated Prx1 (5 μM) increased fluorescence intensity with a rate dependent on GSH concentration. In contrast to the observed S-nitrosated Prx1C83C173S (Figure 1), S-nitrosated Prx1 recovered only 51% of the initial fluorescence intensity of reduced Prx1 (compare Figure 4a,b). Accordingly, the fluorescence increase of S-nitrosated Prx1 with time fitted to a single-exponential function, providing the *k*_obs_ values and indicating that only one of the Prx1 S-nitrosothiol group was denitrosated by GSH under the tested experimental conditions. The plot of *k*_obs_ values *vs* GSH concentration showed a linear dependency (Figure 4c) and the slope provided the second-order rate constant for Prx1 denitrosation by GSH (k = 14.4 ± 0.4) M^−1^. s^−1^ at pH 7.4 and 25 °C. To be confident in the second-order rate constant determined, we also followed the reaction of S-nitrosated Prx1C83SC173S with GSH by fluorescence increasing with time, which fitted to a single-exponential function (Figure S2). The determined *k*_obs_ values vs. GSH concentration provided a second-order rate constant value ((14.5 ± 1.6) M^−1^. s^−1^) (Figure S2), which was practically the same value obtained with Prx1 (Figure 4c). These results establish that Prx1 denitrosation by GSH occurs at the peroxidatic residue (Cys^52^) under the tested experimental conditions.

### 3.3. Interaction of Prx1 with DNIC-GS

As mentioned above, we were interested in examining whether the DNIC-GS was able to nitrosate Prx1 and with which efficiency. In solution, DNIC-GS exists in equilibrium between the binuclear (B-DNIC) and the mononuclear (M-DNIC) forms (reaction 1) [42,50,51]. Here, we used solutions at pH 7.4 containing about 93–95% B-DNIC form (diamagnetic, EPR silent) and 5–7% M-DNIC form (paramagnetic, EPR active) [42,52] that are referred to as DNIC-GS for simplicity. The concentration of these mixtures is reported in terms of the iron center.

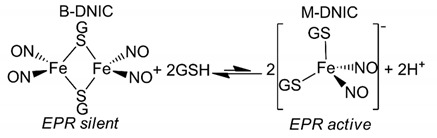
(1)

We first attempted to follow the reaction between DNIC-GS (60 μM) and reduced wild-type Prx1 (30, 60 or 120 μM) by fluorescence but the results are unsatisfactory due to two main reasons. DNIC has a strong light absorption in the UV region [42,51,53] and attempts to separate the iron complex from the protein by gel filtration or cut-off filters after reaction were unsuccessful. The latter result indicates the binding of Prx1 to DNIC-GS. To confirm such an indication, we decided to examine the incubation mixtures by EPR spectroscopy.

**Figure 5:**
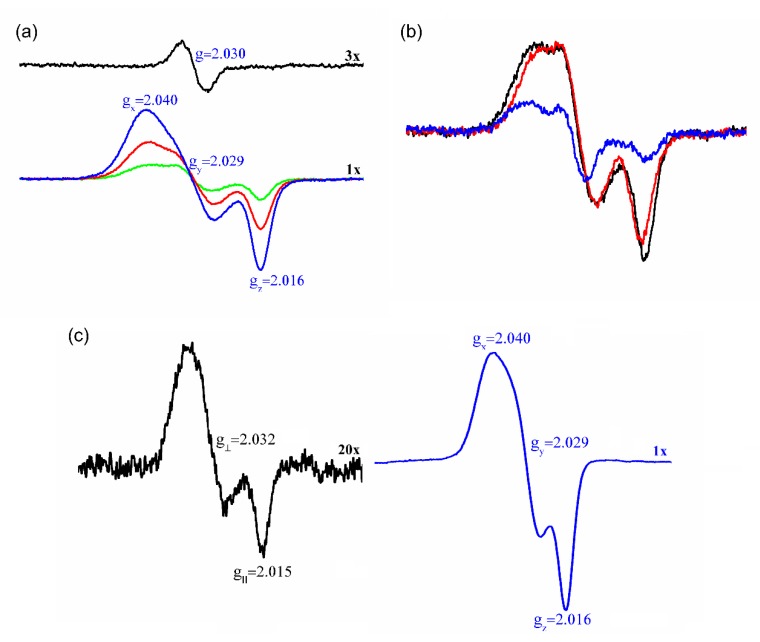
EPR detection of dinitrosyl-iron complex (DNIC)-Prx1 complexes. (**a**) Representative EPR spectra of DNIC-GS (60 μM) before (black trace) and 30 min after addition of 30 (green trace), 60 (red trace) or 120 μM (blue trace) Prx1 scanned at room temperature. (**b**) Representative EPR spectra of DNIC-GS (60 μM) 30 min after addition of 30 μM of wild-type Prx1 (black trace), Prx1C83SC173S (red trace) or Prx1C52S (blue trace) scanned at room temperature. (**c**) Time-dependent 77K EPR spectrum of DNIC-GS (60 μM) plus Prx1 (60 μM) after 2 (black trace) or 30 min (blue trace) incubation. Aliquots of the incubation were removed at the specified times and frozen to stop the interaction and the EPR spectrum acquired at 77 K. All the incubations were performed in phosphate buffer (50 mM) containing DTPA (0.1 mM), pH 7.4 (final) at 25 °C.

**Figure 6:**
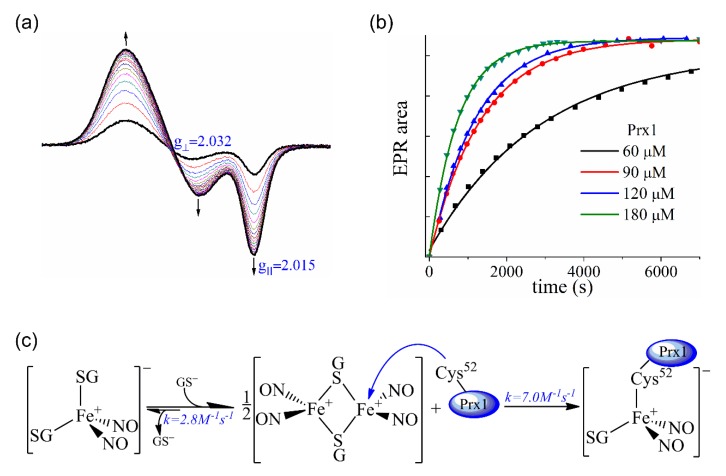
Prx1 Cys^52^ binding to DNIC (**a**) Representative changes in the EPR spectrum with time for the reaction between Prx1 (180 μM) and DNIC-GS (60 μM). (**b**) EPR signal area vs time for the reaction between DNIC-GS (60 μM) and Prx1 (60-180 μM). All the incubations and spectra were performed in phosphate buffer (50 mM) containing DTPA (0.1 mM), pH 7.4 (final) at 25 °C. (**c**) Schematic representation of the reaction between Prx1 peroxidatic Cys and DNIC-GS.

As expected, the room temperature EPR spectra of DNIC-GS exhibited the characteristic single symmetrical line (isotropic spectra) at g = 2.03 (Figure 5a, black trace) [42]. In contrast, incubations of DNIC-GS with Prx1 exhibited anisotropic EPR spectra with rhombic symmetry (g_x_ = 2.040; g_y_ = 2.029; g_z_ = 2.015) in both liquid (Figure 5a) and frozen states (Figure 5c, blue trace). The increased anisotropy at room temperature indicates a slower rotation rate caused by the coordination of a high molecular weight molecule, such as Prx1, to the paramagnetic M-DNIC at pH 7.4. The EPR signal area increased with Prx1 concentration (Figure 6a), indicating that Prx1 displaces the equilibrium between the diamagnetic B-DNIC and the paramagnetic M-DNIC complex (Equation (1)) to the latter form. Notably, Prx2 was also able to displace the GS ligand from DNIC-GS (Figure S3). DNIC-Prx1 and DNIC-Prx2 were stable for at least 5h at pH 7.4.

### 3.4. Prx1 Residues Involved in the Coordination to DNIC-GS

Since DNIC-GS and Prx1 interaction resulted in a DNIC of high molecular weight (DNIC-Prx1), it was relevant to determine which Prx1 residues bind to the iron center. To evaluate the involvement of Cys residues, we determined the thiol contents of Prx1 before and after reaction with excess of DNIC-GS (4-fold) for 30 min at pH 7.4, 25 °C. Reduced Prx1 usually contained 3.5 ± 0.3 thiol/protein, while the DNIC-GS-treated enzyme presented 2.7 ± 0.3 thiol/protein. Therefore, approximately 1.0 thiol/protein disappeared after DNIC-GS treatment. In order to identify the bound Cys residue, Prx1C52S and Prx1C83SC173S mutants (30, 60 or 120 μM) were incubated with DNIC-GS (60 μM) for 30 min at 25 °C and the EPR spectra of the mixtures were scanned. Similar to that observed with Prx1 (Figure 5a), both mutants shifted the DNIC-GS equilibrium to form paramagnetic species (Figure S3). The EPR signal area and the shape of DNIC-Prx1C83SC173S complexes were quite similar to those obtained with Prx1 (Figure 5b, black and red traces). In contrast, Prx1C52S resulted in an 82% decrease in the EPR signal area as compared to Prx1 (Figure 5b, blue trace). Similarly, when Prx1 was oxidized with an equivalent concentration of H_2_O_2_ and then incubated with DNIC-GS, the EPR signal area was reduced by 80%. These data show that the peroxidatic cysteine (Cys^52^) is the most important residue involved in DNIC-Prx1 formation. However, Cys^52^ absence or oxidation did not result in the complete disappearance of the anisotropic EPR signal, indicating that another Prx1 residue is also able to coordinate to DNIC.

Since His is a common iron ligand in biology [54], Prx1 was treated with diethylpyrocarbonate (DEP) under conditions of preferential His modification [39,40]. The incubation of DEP-treated Prx1 with DNIC-GS resulted in an EPR signal of axial symmetry (g_┴_ = 2.03 and g_║_ = 2.014) at both room temperature (Figure S4) and 77 K (Figure S4, Insert). Axial symmetry is a characteristic of DNIC with two equivalent ligands, indicating that the EPR signal shown in Figure S4 is due to a DNIC containing two ligands coordinated by the thiol group (GSH and the Prx1 peroxidatic cysteine; [Fe(NO)_2_(GS)(Cys^52^-Prx1)]^−^). The lack of a rhombic symmetry contribution in the signal resulting from DEP-treated Prx1 indicated that a Prx1 His residue is also able to coordinate to DNIC ([Fe(NO)_2_(GS)(His-Prx1)]^−^) and is responsible for the rhombic EPR signal (g_x_ = 2.040; g_y_ = 2.029; g_z_ = 2.015). In another set of experiments, we pre-treated Prx1 with thiol alkylating agents (NEM and IAM) before interaction with DNIC-GS. Both agents precluded the formation of DNIC-Prx1 complexes (Figure S5), indicating that alkylation of Prx1 Cys makes the Prx1 His residue inaccessible to DNIC-GS.

We did not determine which Prx1 His residue coordinates to DNIC because a comparison of the relative binding affinity of Prx1 Cys^52^ and Prx1 His to the complex indicated that Prx1 Cys^52^ is the preferential DNIC ligand. Indeed, time-dependent incubation of Prx1 with DNIC-GS with freezing of the samples to stop the processes showed that after 2 min, the EPR signal is essentially of axial symmetry but it is of rhombic symmetry after 30 min incubation (Figure 5c). Additionally, as Prx1 concentration increases over DNIC-GS concentration, the EPR signal acquires a more pronounced axial symmetry (Figure 5a). In other words, if DNIC-GS is not in excess over Prx1, the axial symmetry component of the EPR spectrum increases, corroborating that Prx1 Cys^52^ residue as the preferential DNIC ligand. In agreement, after treatment with one or two equivalents of DNIC-GS, Prx1 loses its peroxidase activity (Figure S6).

### 3.5. Kinetics of the Cys^52^-Px1 Binding to DNIC-GS

Taking advantage of the greater binding affinity of the peroxidatic Cys toward DNIC-GS, the variation of the axial EPR signal area (Figure 6a) with time was used to calculate the second-order rate constant for [Fe(NO)_2_(GS)(Cys^52^-Prx1)]^−^ formation. The DNIC-GS concentration was fixed at 60 μM and Px1 concentration varied from 60 to 180 μM. Notably, the EPR signal area reaches the same plateau with time for all Prx1 concentrations (60–180 μM) (Figure 6b). Quantification of the EPR signal area at the plateau showed that DNIC-GS (60 μM) is fully converted into DNIC-Prx1 (60 μM). The plot of EPR signal area vs. time fitted to a single exponential curve (Figure 6b). The calculated *k*_obs_ values plotted against Prx1 concentration exhibited a linear dependency (data not shown) from which the slope provided the second-order rate constant for [Fe(NO)_2_(GS)(Cys^52^-Prx1)]^−^ formation as 7.0 ± 0.4 M^−1^. s^−1^ at pH 7.4 and 25 °C.

As mentioned before, the DNIC-GS solutions used here contain about 93–95% B-DNIC-GS and 5–7% of M-DNIC-GS [42], therefore we wondered which of these species is the Prx1 main target for binding. Additionally, if M-DNIC-GS is the Prx1 target, the rate of Prx1 binding would be limited by the B-DNIC-GS to M-DNIC-GS conversion. Therefore, we decided to calculate the rate of B-DNIC-GS to M-DNIC-GS conversion. For this propose, a fixed concentration of DNIC-GS (0.3 mM) was mixed to an excess of GSH (6-26 mM) at pH 7.4 (200 mM sodium phosphate) and 25 °C using stopped-flow equipment. Under these conditions, B-DNIC-GS (ε_310nm_ = 9200 and ε_360nm_ = 7400 M^−1^. s^−1^) shifts to M-DNIC-GS (ε_400nm_ = 4,700 M^−1^. cm^−1^) [42]. The increase in absorption at 400 nm was followed as a function of time and fitted to a single exponential function (Figure S6), similarly to that previously observed for DNIC-Cys [43]. The *k*_obs_ values vs. thiol concentration showed a linear dependence and the slope provided the second-order rate constant for B-DNIC-GS to M-DNIC-GS conversion as k = (2.8 ± 0.1) M^−1^. s^−1^ at pH 7.4 and 25 °C (Figure S7). According to this, the rate of [Fe(NO)_2_(GS)(Cys^52^-Prx1)]^−^ formation is 2.5-fold faster than the rate for B-DNIC-GS to M-DNIC-GS conversion, indicating that B-DNIC-GS is the main target for Prx1 binding (Figure 6c).

### 3.6. Evaluation of DNIC-GS and DNIC-Prx1 as Nitrosating Agents

Up to this point, the results show that the interaction between DNIC-GS and Prx1 results in the quantitative formation of DNIC-Prx1 complexes (Figure 6b). Since our initial aim was to examine whether DNIC promotes Prx1 S-nitrosation, we also investigated the capability of DNIC-GS and DNIC-Prx1 to mediate the S-nitrosation of coordinated and non-coordinated Prx1. To this end, Prx1 was treated with stoichiometric (1Prx1:1DNIC), substoichiometric (3Prx1:1DNIC) and, excess (1Prx1:4DNIC) of DNIC-GS for 1 h at 25 °C. The low molecular weight molecules were washed away using cut-off filters (10 kDa) and Prx1 (Bradford method), DNIC (EPR) and nitric oxide (NO-Analyzer) levels were determined. Under all the investigated conditions, the ratio of DNIC concentration to nitric oxide concentration was maintained as 1 to 2, demonstrating that Prx1 S-nitrosation does not occur under all of these experimental conditions.

## 4. Discussion

Protein S-nitrosation is an important consequence of NO^^●^^ metabolism with implications in multiple pathologies [1]. After the proposal that protein S-nitrosation may mediate NO^^●^^ signaling, extensive efforts were directed to uncover targets of S-nitrosation in cells through either targeted or proteome-wide approaches [6]. These investigations provided a myriad of information; however, in most cases, quantification of the extent of protein nitrosation is lacking. This point is important because, in cells, protein S-nitrosation occurs in parallel with other NO^^●^^-dependent modifications, such as protein oxidation, protein nitration, hemeprotein nitrosylation, and DNICs formation. The relative importance of each modification and of each modified target depends on target concentration and the kinetics of the involved reactions, arguing for the importance of kinetic studies to advance our understanding of biological responses [5,55].

In this work, we investigated the kinetics of the reactions of Prx1 and its mutants (Prx1C52S and Prx1C83SC173S) with GSNO, the most used agent to trigger protein S-nitrosation in cells, and with DNIC-GS, as an example of DNICs supposedly acting as an S-nitrosating agent [9,56,57]. To study the kinetics of the reaction between Prx1 and GSNO, we first showed that reduced Prx1C83SC173S presented a time-dependent loss of its intrinsic fluorescence while reacting with GSNO (Figure 1) to become nitrosated, as confirmed by thiol/nitrosothiol quantification (Table 1). Addition of GSH excess to nitrosated Prx1C83SC173S led to the recovery of the intrinsic fluorescence of the reduced enzyme, indicating the reversibility of the process and its adequacy for kinetic studies. Under pseudo-first-order conditions the profile of the kinetic curves obtained with Prx1C83SC173S (5 µM) and various GSNO concentrations fitted to a single exponential (Figure 2a), confirming nitrosation of a single Cys residue, likely the peroxidatic one (Cys^52^). These experiments provided the second-order rate constant of the reaction between Prx1C83SC173S and GSNO as 15.4 ± 0.4 M^−1^. s^−1^ (Figure 2b). In contrast, the profile of the kinetic curves obtained with Prx1 (wild type) fitted to a double exponential function (Figure 2c), indicating the reaction of two Cys residues with quite different reactivity towards GSNO. The most reactive Prx1 residue reacted with GSNO with the same second-order rate constant determined for Prx1C83SC173S, confirming that it was the peroxidatic residue. To establish that Prx1Cys^83^ was the second reactive Cys residue instead of Cys^173^, we performed complementary kinetic analysis between oxidized Prx1 (Prx1-Cys^52^-Cys^173^-Prx1) and GSNO (Figure 3b), observing that the slower exponential decay was maintained. With these experiments, we were able to determine the second-order rate constant values of the reaction between GSNO and the Prx1 peroxidatic residue ($k_{+NO}^{Cys52}$ = 15.4 ± 0.4 M^−1^. s^−1^) and the Prx1 Cys^83^ residue ($k_{+NO}^{Cys83}$ = 1.7 ± 0.4 M^−1^. s^−1^) at pH 7.4 and 25 °C.

It was previously reported that the spontaneous denitrosation of Prx1 nitrosated by CysNO was relatively rapid and probably occurred by the attack of the resolving Cys residue (Cys^173^) on the nitrosated Cys^52^ residue (Prx1-Cys^52^NO), based on an observed time-dependent increase in the disulfide Prx1 dimer (Prx1-Cys^52^-Cys^173^-Prx1) in non-reducing SDS-PAGE experiments [23]. Using GSNO as the nitrosating agent, we also performed non-reducing SDS-PAGE experiments but did not observe a time-dependent increase in disulfide Prx1 dimer formation (Figure S1). Some dimer formation occurred but it was quite rapid and did not increase with time, indicating that Prx1 oxidation occurred due to contaminant H_2_O_2_ being present in the buffer and/or reagents. Additionally, we were able to purify nitrosated Prx1 from excess reagents and study the kinetics of its spontaneous and GSH-promoted denitrosation (Figure 4). The spontaneous denitrosation of nitrosated Prx1 followed first-order kinetics (Figure 4a, blue trace), the rate constant of which was determined as $k_{-NO}^{}$ = (7.5 ± 0.5) × 10^−4^ s^−1^ at pH 7.4 and 25 °C (Figure 4a, blue trace). The profile of the kinetic curves of the reaction of nitrosated Prx1 with GSH fitted to single exponentials (Figure 4a), suggesting that it occurs mainly through a transnitrosation reaction instead of a mixed process involving both transnitrosation (reaction 2) and formation of a mixed disulfide (Prx1Cys^52^-SG) (reaction 3). Transnitrosation is also favored by the value determined for the second-order rate constant of the reaction between Prx1-Cys^52^NO and GSH ($k_{-NO}^{GSH}$ = 14.4 ± 0.4 M^−1^. s^−1^) (Figure 4b). Indeed, the value of this second-order rate constant is close to that determined for Prx1Cys^52^ nitrosation by GSNO ($k_{+NO}^{Cys52}$ = 15.4 ± 0.4 M^−1^. s^−1^), following the trend of transnitrosation reactions having equilibrium constant values approaching 1.0 [6]. In the case of proteins, this trend should hold in the absence of major structural protein changes. The fact that we did not observe a measurable denitrosation rate of Prx1Cys^83^ residue by GSH (see Results) suggests that GSNO concentrations sufficiently high to promote considerable nitrosation of such residue lead to a more extensive Prx1 structural modification.

(2) $${\mathrm{Prx}‐\mathrm{Cys}}^{52}\mathrm{NO}+\mathrm{GSH}⇌{\mathrm{Prx}‐\mathrm{Cys}}^{52}+\mathrm{GSNO}$$

(3) $${\mathrm{Prx}‐\mathrm{Cys}}^{52}\mathrm{NO}+\mathrm{GSH}\to {\mathrm{Prx}‐\mathrm{Cys}}^{52}\mathrm{SG}+\mathrm{HNO}$$

Our kinetic studies of Prx1 and Prx1C83SC173S nitrosation by GSNO indicate that in most cellular environments, in which the expected concentrations of GSNO and GSH are in the range of µM and mM, respectively, nitrosation of the peroxidatic Prx1 residue is more likely to occur. However, this modification is likely to be transient, since GSH is able to repair it as efficiently as GSNO forms it. Although Trx was reported to catalyze S-nitrosation and S-denitrosation reactions [22,58,59], Trx concentration is extremely low as compared to that of GSH and the enzyme cannot alter the equilibrium position of the reactions [6]. These considerations suggest a relatively low biological relevance of Prx1 nitrosation by GSNO in cells.

The investigation of the hypothetical nitrosation of Prx1 and mutants by DNIC-GS rendered a quite surprising discovery. We clearly demonstrated by EPR experiments at room temperature that Prx1 and the mutants (Prx1C52S and Prx1C83SC173S) are able to displace one of the GSH ligands from the DNIC-GS complex, forming high molecular weight DNIC-Prx1complexes (Figure 5). Interestingly, Prx2 was also able to form DNIC-Prx2 complexes (Figure S3). Both DNIC-Prx1 and DNIC-Prx2 complexes are stable for at least 5h at room temperature.

To identify the Prx1 residue involved in the coordination to DNIC, we performed and analyzed several experiments. We determined that about 1.0 thiol disappears upon Prx1 reaction with excess DNIC-GS. Also, we compared the shape and area of the EPR spectra obtained from the reaction of DNIC-GS with Prx1with those obtained for the C83SC173S and C52S mutants (Figure 5b; Figure S3) and for Prx1 pre-oxidized with one H_2_O_2_ equivalent (not shown). These experiments showed that the peroxidatic cysteine (Cys^52^) is the most important residue involved in DNIC-Prx1 complex formation but another residue is also involved and responsible for about 20% of the observed final spectrum of DNIC-Prx1. Additional experiments with DEP-pretreated Prx1 and analysis of the shape of the resulting EPR spectrum led us to establish His as the second Prx1 residue able to bind DNIC (Figure S4). The identity of this His residue was not pursued because analysis of the shape of the EPR spectra with increased Prx1 over DNIC-GS concentration (Figure 5a) and with time (Figure 5c) indicated that Prx1 Cys^52^ is the preferential DNIC ligand.

To propose a mechanism for the interaction between Prx1 and DNIC-GS to produce preferentially DNIC-Cys^52^Prx1 ([Fe(NO)_2_(GS)(Cys^52^-Prx1)]^−^), we determined the second-order rate constant of complex formation as k = (7.0 ± 0.4) M^−1^. s^−1^ at pH 7.4 and 25 °C (Figure 6a,b). We also considered the fact that Prx1 reacted quantitatively with all available DNIC-GS (Figure 5a, Figure 6a,b), which is initially an equilibrated mixture of 93–95% B-DNIC-GS form (EPR silent) and 5–7% M-DNIC-GS form (EPR active). Therefore, Prx1 shifts the equilibrium between the B-DNIC and M-DNIC to the latter form (reaction 1). The second-order rate constant for B-DNIC-GS to M-DNIC-GS conversion was determined as k = (2.8 ± 0.1) M^−1^. s^−1^ at pH 7.4 and 25 °C (Figure S6). Since the rate of [Fe(NO)_2_(GS)(Cys^52^-Prx1)]^−^ formation is 2.5-fold faster than the rate for B-DNIC-GS to M-DNIC-GS conversion, we propose that B-DNIC-GS is the main target for Prx1 attack (Figure 6c). This may be relevant under physiological conditions, in which the majority of DNIC complexes are likely to be present in diamagnetic forms.

The formation of DNIC-protein complexes was previously described for bovine serum albumin [9] and for proteins of the glutathione transferase (GST) family [60,61]. The latter were extensively investigated by Ricci and co-workers, who showed that these enzymes, in particular the Tyr GSTs, have an extremely high affinity for DNIC, forming DNIC-GST complexes, whose dissociation constant attain the range of 10^−9^ to 10^−12^ M [62]. In cells, Prx1 probably cannot compete with GSTs for DNIC complexes because of the low second-order rate constant for its formation. Nevertheless, the demonstration that Prx1 coordinates to DNIC opens such a possibility for other thiol proteins.

Finally, we also showed that DNIC-GS and DNIC-Prx1 do not cause nitrosation of free Px1 or DNIC bound Prx1 and such inability likely holds for other proteins. Therefore, the fact that activated macrophages under anoxia produce DNICs and nitrosothiols in parallel [10,57] is unlikely due to the ability of DNICs to nitrosate low and/or high molecular weight thiols. A more likely possibility is provided by the recent demonstration that during DNIC-GS and DNIC-Cys formation, a concomitant generation of the corresponding thiyl radicals occurs [53]. Consequently, in a NO^^●^^ rich environment, such in activated macrophages under anoxia, formation of DNICs complexes would occur in parallel with thiyl radicals, which recombine with NO^^●^^ at diffusion controlled rates, leading to concomitant formation of nitrosothiols.

## 5. Conclusions

Our study shows that the Prx1 peroxidatic Cys is the more reactive residue toward S-nitrosation by GSNO and toward formation of DNIC-Prx1 by DNIC-GS. Both processes impair the peroxidase activity of 2-Cys Prxs, disrupting their antioxidant and signaling functions. The Prx1 reactions with both GSNO and DNIC-GS were not particularly rapid and, therefore, unlikely to be relevant in cells because of the many competitive reactions that occur under physiological conditions. Nevertheless, both processes occurred with measurable rates and may be faster when mediated by nitrosating agents other than GSNO and DNIC complexes other than DNIC-GS. Indeed, nitrosation of Prx1 and Prx2 has been demonstrated in cells [20,21,22,23,24], whereas high molecular weight DNIC complexes were investigated in tissue extracts only for GSTs [63] to the best of our knowledge. Therefore, the nature of the high molecular weight DNIC complexes formed in cells overproducing NO^^●^^ deserves further investigation.

## Figures and Tables

**Table 1:** Summary of thiol and nitrosothiol contents in reduced and GSNO-treated Prx1C83SC173S and Prx1.

Protein	Reduced	Pre-Treated with GSNO	
	R-SH/Protein	R-SH/Protein	R-SNO/Protein
Prx1C83SC173S	1.8 ± 0.2	1.0 ± 0.2	0.7 ± 0.1
Prx1	3.5 ± 0.3	1.1 ± 0.2	1.8 ± 0.3

## Supplementary Materials

The following are available online at https://www.mdpi.com/2076-3921/9/4/276/s1, Figure S1: Representative non-reducing SDS gel of reduced Prx1 (2.2 mg) incubated with GSNO (80-fold molar excess) for different times, Figure S2: Determination of the second-order rate constant of the reaction between Prx1C83SC173S-Cys^52^NO and GSH, Figure S3: EPR analysis of Prx2 and Prx1 mutants binding to DNIC, Figure S4: Representative EPR spectrum of DNIC-GS (60 mM) 30 min after addition of Prx1 pre-treated with DEP (60 mM) at room temperature, Figure S5: Representative EPR spectrum of DNIC-GS (60 mM) 30 min after addition of Prx1 pre-treated with alkylating agents (NEM and IAM) at room temperature, Figure S6: Prx1 binding to DNIC inactivates the peroxidase activity, Figure S7: Kinetics of the conversion of B-DNIC-GS into M-DNIC-GS.
