# Supplementary material for: The Peroxidatic Thiol of Peroxiredoxin 1 is Nitrosated by Nitrosoglutathione but Coordinates to the Dinitrosyl Iron Complex of Glutathione
Source: Antioxidants (Basel). 2020 Mar 25;9(4):276. doi: 10.3390/antiox9040276 (PMC7222187; doi:10.3390/antiox9040276)
Supplement: Supplementary file 1 [file antioxidants-09-00276-s001.pdf]

antioxidants

The peroxidatic thiol of peroxiredoxin 1 is nitrosated by nitrosoglutathione but coordinates to the dinitrosyl iron complex of glutathione

Daniela R. Truzzi [1,*], Simone V. Alves [2], Luis E. S. Netto [2] and Ohara Augusto[1]

1 Departamento de Bioquímica, Instituto de Química, Universidade de São Paulo, São Paulo, 05508-000, Brazil; dtruzzi@iq.usp.br (D.R.T.); oaugusto@iq.usp.br (O.A.)
2 Departamento de Genética e Biologia Evolutiva, Instituto de Biociências, Universidade de São Paulo, São Paulo, 05508-090, Brazil; svidigal@ib.usp.br (S.V.A.); nettoles@ib.usp.br (L.E.S.N.)
* Correspondence: dtruzzi@iq.usp.br

Supplementary Material

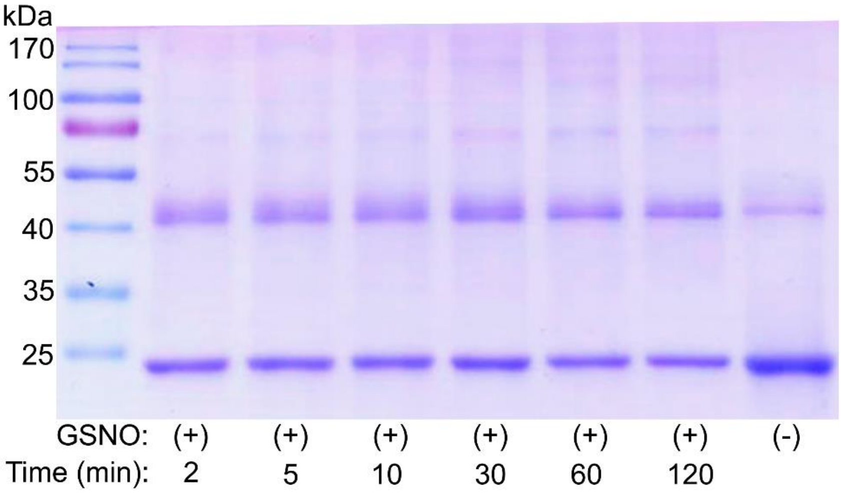

Figure 1. Representative non-reducing SDS gel of reduced wild-type Prx1 (2.2µg) incubated with GSNO (80-fold molar excess) for different times in phosphate buffer (50 mM) containing DTPA (0.1 mM), pH 7.4, at 25 °C.

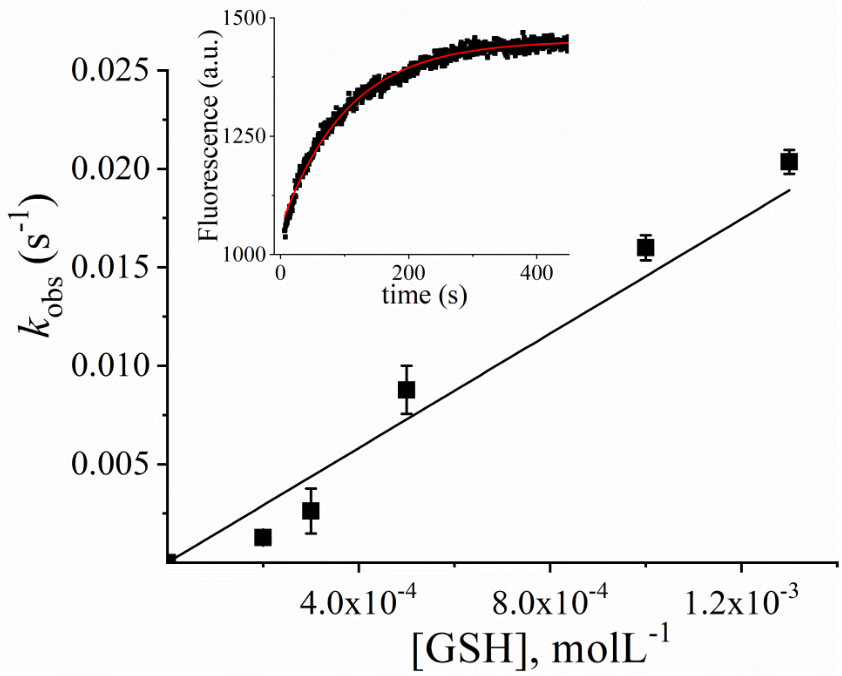

Figure 2. Determination of the second-order rate constant of the reaction between Prx1C83SC173S-Cys[52]NO and GSH. Pseudo-first-order rate constants (k_{obs}) were plotted against glutathione concentration and the second-order rate constant obtained from the slope. *Insert* temporal fluorescence changes. All the experiments were performed phosphate buffer (50 mM) containing DTPA (0.1 mM), pH 7.4, at 25 °C in a Hitachi fluorimeter.

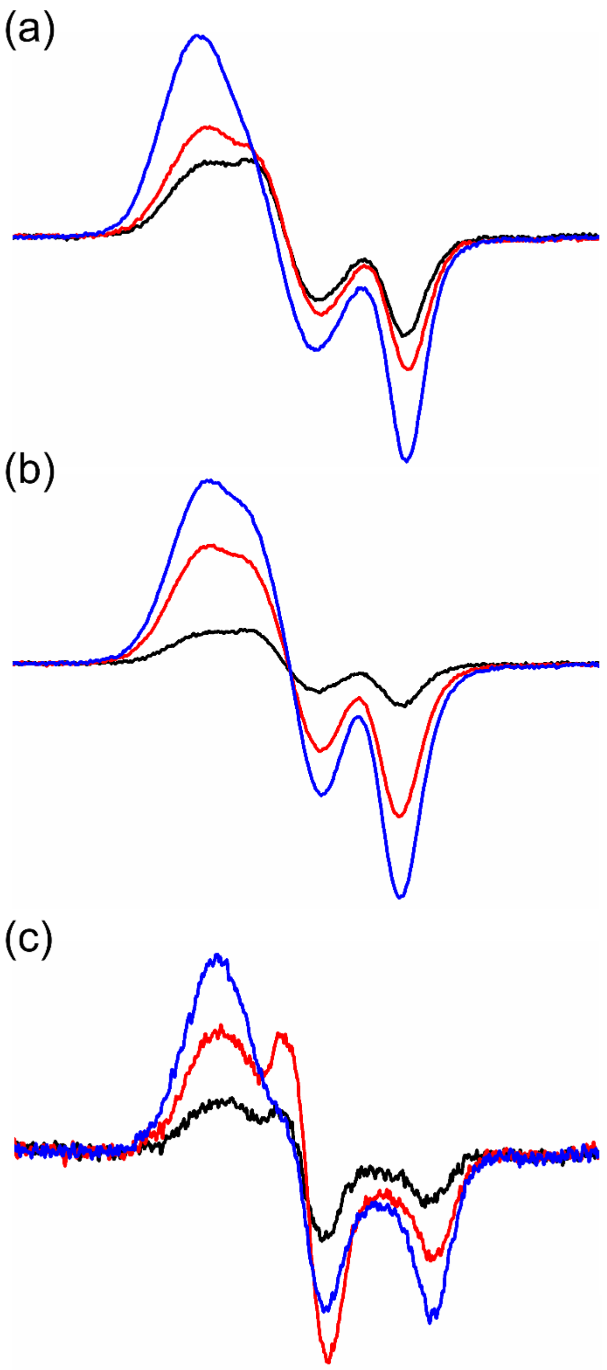

Figure 3. EPR analysis of Prx2 and Prx1 mutants binding to DNIC. (**a**) Representative EPR spectra of 30 µM (black trace), 60 µM (red trace) or 120 µM (blue trace) of Prx2 30 min after addition of DNIC-GS (60 µM) at room temperature. (**b**) Representative EPR spectra of 30 µM (black trace), 60 µM (red trace) or 120 µM (blue trace) of Prx1C83SC173S 30 min after addition of DNIC-GS (60 µM) at room temperature. (**c**) Representative EPR spectra of 30µM (black trace), 60µM (red trace) or 120 µM (blue trace) of Prx1C52S 30 min after addition of DNIC-GS (60 µM) at room temperature. All the incubations were performed in phosphate buffer (50 mM) containing DTPA (0.1 mM), final pH 7.4, at 25 °C.

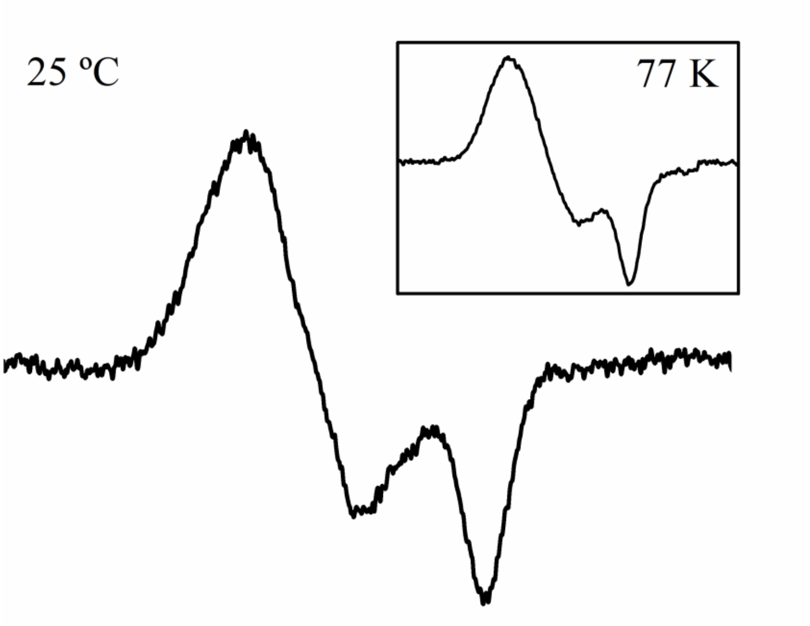

Figure 4. Representative EPR spectrum of DNIC-GS (60 μM) 30 min after addition of Prx1 pre-treated with DEP (60 μM) measured at room temperature. Insert EPR spectrum of the same sample acquired at 77 K.

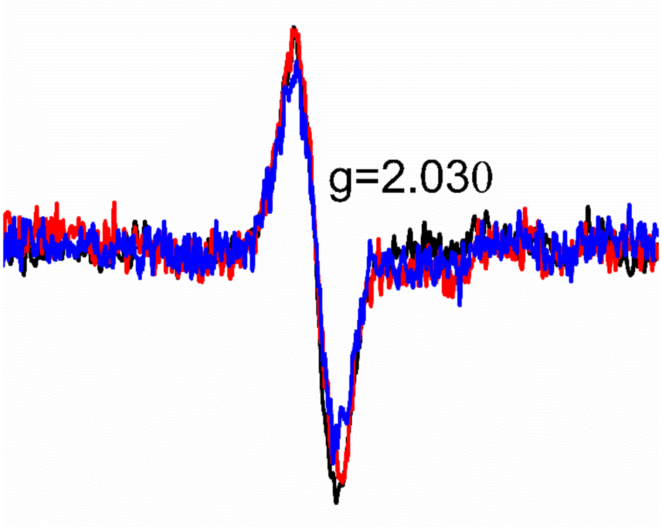

Figure 5. Representative EPR spectrum of DNIC-GS (60 μM) (black trace) and of DNIC-GS (60 μM) 30 min after addition of Prx1 (60 μM) pre-treated with the alkylating agent NEM (red trace) or the alkylating agent IAM (blue trace) at room temperature. All the incubations were performed in phosphate buffer (50 mM) containing DTPA (0.1 mM), final pH 7.4, at 25 °C.

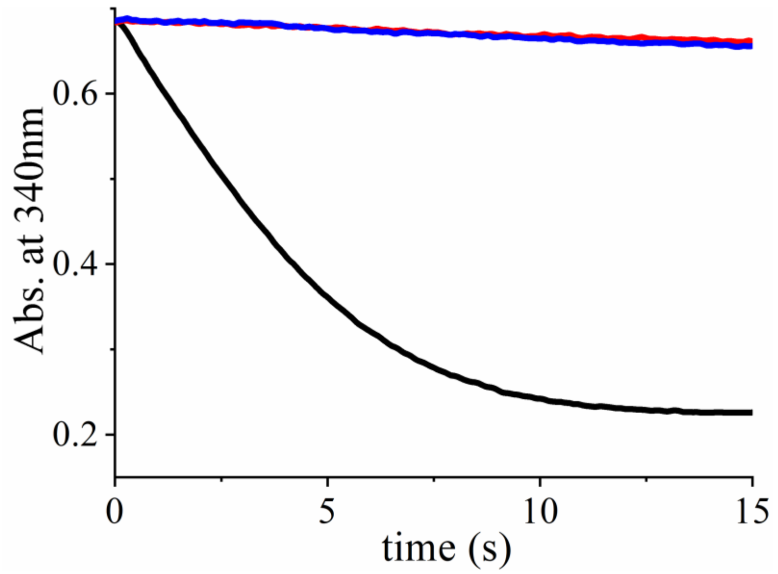

Figure 6. Prx1 binding to DNIC inactivates the peroxidase activity. Wild-type Prx1 activity before (black trace) and after reaction with DNIC-GS (blue and red traces). Prx1 (60 µM) was incubated with 60 µM (blue trace) or 120 µM (red trace) of DNIC-GS for 30 min and the low molecular weight contaminants were removed by ultrafiltration (cut-off filter 10-kDa) and protein concentration quantified by the Bradford method. NADPH oxidation was monitored at 340 nm in a mixture containing 3 µM yTrx, 1.5 µM yTrxR, 100 µM NADPH, 200 µM hydrogen peroxide and 0.5 µM of Prx1 or DNIC-Prx1 in Hepes-NaOH (50 mM) containing DTPA (0.1 mM), pH 7.0 at 30 °C. The data shown are representative of at least three independent experiments.

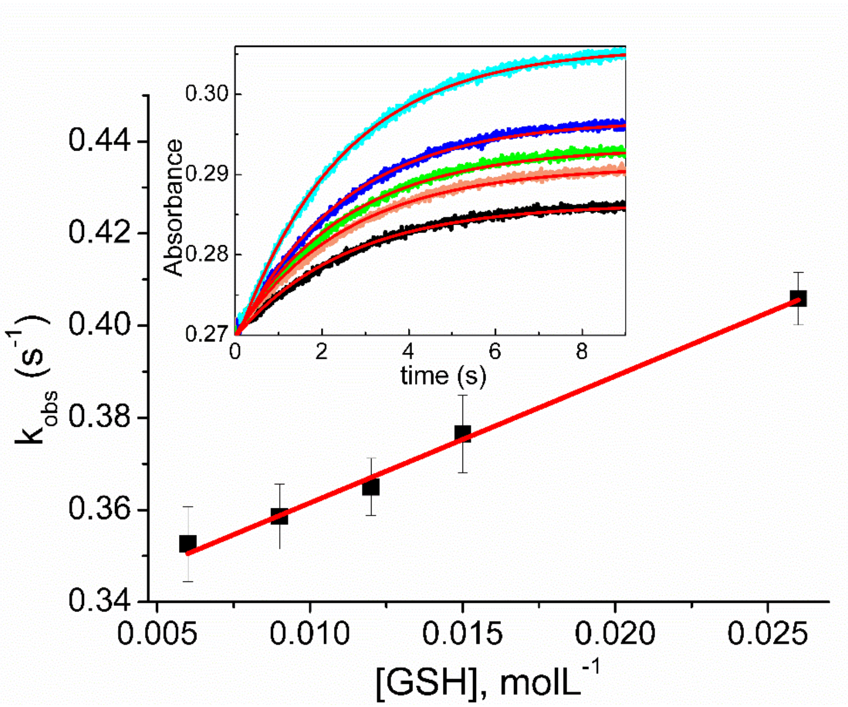

Figure 7. Kinetics of conversion of B-DNIC-GS into M-DNIC-GS. The plot k_{obs} monitored at 400 nm *vs* concentration of GSH at pH 7.4 (200 mM sodium phosphate) and 25°C. *Insert* temporal absorbance changes at 400 nm. The red traces display the fits of these data to single exponential functions to give k_{obs}. .
